# Relationships between autistic traits, self‐efficacy, and internalizing symptoms in a nonclinical sample of Indian early adolescents

**DOI:** 10.1002/brb3.3646

**Published:** 2024-08-12

**Authors:** Megan Cherewick, Christina M. Cruz, Priscilla Giri, Michael Matergia, Roshan P. Rai

**Affiliations:** ^1^ Department of Community & Behavioral Health, Colorado School of Public Health University of Colorado Anschutz Medical Campus Aurora Colorado USA; ^2^ Department of Psychiatry University of North Carolina at Chapel Hill School of Medicine Chapel Hill North Carolina USA; ^3^ School Psychology Program University of North Carolina at Chapel Hill School of Education Chapel Hill North Carolina USA; ^4^ Darjeeling Ladenla Road Prerna Darjeeling West Bengal India; ^5^ Broadleaf Health & Education Alliance Stroudsburg Pennsylvania USA; ^6^ Center for Global Health Colorado School of Public Health Aurora Colorado USA

**Keywords:** adolescence, autism, LMIC, neurodiversity, self‐efficacy

## Abstract

**Introduction:**

Having more “autistic traits” is associated with an increased risk of mental health conditions. However, few studies have examined autistic traits in nonclinical samples. This study aims to analyze the relationship between autistic traits and internalizing symptoms among early adolescents and to examine the moderating effect of self‐efficacy.

**Methods:**

Survey data were collected from early adolescents ages 10–14 living in Darjeeling, India (*n* = 274) to assess autistic traits, self‐efficacy, and internalizing symptoms.

**Results:**

Higher internalizing symptoms were significantly associated with a higher report of autistic traits. Academic, social, and emotional dimensions of self‐efficacy moderated the relationship between autistic traits and internalizing symptoms.

**Conclusion:**

The moderation effects between social and emotional self‐efficacy among youth with high versus low autism trait scores suggest the need for social‐emotional learning interventions designed for and with neurodivergent youth. Such interventions aim to reduce internalizing symptoms during early adolescence and improve future mental health trajectories.

## Graphical abstract

This study indicates that higher levels of autistic traits are linked to higher levels of internalizing symptoms among early adolescents ages 10‐14. The moderation effects of social and emotional self‐efficacy on internalizing symptoms, observed in youth with varying levels of autistic traits, highlight the necessity for social‐emotional learning interventions that are neurodiversity affirming and inclusive. Such interventions are essential at the population level to improve mental health trajectories.

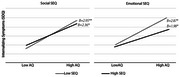

## 1 INTRODUCTION

Autism Spectrum Condition (ASC) has a global prevalence rate estimated to be 1.5–2%, making it one of the most common developmental conditions (Baird et al., 2006; Fombonne, 2009; Lord & Spence, 2006; Lyall et al., 2017). The majority of autistic individuals live in low‐ and middle‐income countries (LMIC), with approximately 2 million autistic youth living in India (Deshmukh et al., 2013; Rahman et al., 2016). Autistic children are at a profound risk for mental health conditions in comparison to typically developing children (Bandura et al., 1999; Sandin et al., 2015; Schönfeld et al., 2016). A new and growing movement led by autistic individuals and advocates calls for a paradigm shift away from deficit‐based models toward a *neurodiversity affirming* framework. This perspective views autism and associated traits as an expected variation in neural development (Johnson, 2017; Lai et al., 2015). Advances in the development of population measures of autism aim to create measurement scales for nonclinical populations that can be administered by nonspecialists. Accordingly, more recent measures are founded on a theoretical paradigm that asserts that individual traits associated with autism are distributed across human populations and, at a threshold level, meet common diagnostic criteria for ASC. Few studies have examined how these new measures can be useful in nonclinical populations to better tailor and target interventions. Such interventions can support autistic and neurodivergent individuals by identifying protective factors that reduce mental health disorders and promote mental wellbeing.

A growing body of evidence supports self‐efficacy as a protective factor that reduces the risk for mental health disorders and promotes mental wellbeing. Self‐efficacy is defined as the perceived ability to complete a desired action, tackle a novel task, or cope with a broad range of stressors (Bandura et al., 1999; Luszczynska et al., 2005). Previous studies have reported that lower self‐efficacy is associated with higher levels of depressive and anxiety symptoms (Comunian, 1989; Ehrenberg et al., 1991; Kashdan & Roberts, 2004; Kwasky & Groh, 2014; Luszczynska et al., 2005). Muris (2001) developed a self‐efficacy questionnaire for children (SEQ‐C) to measure three dimensions of self‐efficacy, including academic, social and emotional self‐efficacy (Muris, 2001). Academic self‐efficacy refers to an individual's perceived ability to control learning behaviors, master subject content, and meet academic expectations; social self‐efficacy refers to an individual's perceived ability to be assertive and form relationships with peers; and emotional self‐efficacy refers to an individual's perceived ability to cope with negative emotions (Muris, 2001).

While the majority of autism interventions target early childhood, a second period of heightened neuroplasticity is triggered by the onset of puberty, creating a sensitive window for social‐emotional learning (Cherewick, 2023; Mottron et al., 2014). During adolescence, self‐efficacy develops through reciprocal relationships between intrapersonal factors (affective, behavioral, and cognitive capacities) and social and environmental factors. Self‐efficacy influences individual behavior patterns and is shaped by social and environmental conditions. Early adolescence is a particularly opportune time to deliver interventions aimed at reducing the risk of mental health disorders before clinically significant symptoms increase during middle and later adolescence. This opportunity is especially crucial for neurodivergent and autistic early adolescents. Studies estimate the risk for attempting suicide is more than three times higher for autistic individuals compared to neurotypical individuals, and 8.5 times higher for autistic girls compared to neurotypical girls (Hodgetts et al., 2015; Kogan et al., 2008; Milton & Sims, 2016; Vohra et al., 2017). Autistic adults have argued that a significant cause of these tragic statistics is related to diminished self‐perception, autonomy, and self‐esteem (Hodgetts et al., 2015; Kogan et al., 2008; Milton & Sims, 2016; Vohra et al., 2017). Prevention programs that target self‐efficacy could address these vulnerabilities and improve mental health outcomes.

### 1.1 Study aims and hypotheses

The aims of the current study were to (1) examine the associations between subdomains of autistic traits, self‐efficacy, and internalizing symptoms by sex and (2) to assess the strength of these associations, including the interaction between autistic traits and self‐efficacy, in predicting internalizing symptoms. We hypothesize that (1) subdomains of autistic traits and self‐efficacy will be significantly associated with internalizing symptoms and (2) higher self‐efficacy scores will moderate the relationship between autistic traits and internalizing symptoms, such that higher self‐efficacy scores will attenuate the association between higher autistic traits and internalizing symptoms.

## 2 METHODS

### 2.1 Procedure

The study sample (*n* = 274) comprised urban, peri‐urban, and rural early adolescents in the Darjeeling Himalayas, a district in the state of West Bengal, India. Four schools were recruited to participate in the study based on positive working relationships with the local nongovernmental organization, Darjeeling Ladenla Road Prerna (DLRP). Together with school leaders, DLRP identified classrooms with eligible adolescents. Classrooms were then selected using a random number generator. Within each class, all eligible participants were invited to participate. Eligibility criteria included: (1) age 10–14 upon enrollment, (2) residence in Darjeeling, India, (3) attendance at one of four low‐cost private schools, (4) caregiver written consent, (5) participant verbal assent. Participants were informed that their participation was voluntary, they could withdraw from the study at any time, and they were given a description of the study. Participants completed 45‐min surveys administered after school on school grounds in a private location. Research assistants read each question aloud in Nepali, and participants recorded their responses on a corresponding written survey. Participant responses were then entered into the Research Electronic Data Capture (REDCap) application (Patridge & Bardyn, 2018).

### 2.2 Ethics standard

This study was performed in accordance with the ethical standards in the 1964 Declaration of Helsinki and its later amendments or comparable ethical standards. The study was approved by the Colorado Multiple Institutional Review Board (COMIRB) and the St. Josephs College Ethics Committee in Darjeeling, India: Protocol No. 23–1421. Informed written consent was obtained from all caregivers of adolescents and informed assent obtained from all adolescents included in the study.

### 2.3 Measures

*The Autism Spectrum Quotient (AQ)* is a self‐report measure that consists of 50 statements to assess autistic traits in a general population (Baron‐Cohen et al., 2001). Participants respond a four point Likert scale (1 = definitely agree, 2 = slightly agree, 3 = slightly disagree, 4 = definitely disagree). In concordance with the authors recommendations, the four point scale is reduced to a dichotomous scale by assigning one point to responses of slightly agree or agree in one half of items and one point to responses of disagree or slightly disagree in the other half. The total score ranges from 0 to 50 points, with higher scores indicating greater number of autistic traits. Previous research has demonstrated that individuals with a diagnosis of ASC score higher than nonclinical samples on the total AQ and individual subscales (Baron‐Cohen et al., 2001; Broadbent et al., 2013; Hoekstra et al., 2008; Lau et al., 2013). The test‐retest reliability and internal consistency of the AQ have been previously evaluated as acceptable (Baron‐Cohen et al., 2001; Broadbent et al., 2013; Hoekstra et al., 2008; Lau et al., 2013). The Cronbach's alpha in this sample was α = .75.

*Self‐Efficacy Questionnaire for Children (SEQ)* is a 24‐item questionnaire developed by (Muris, 2001) for use with children and adolescents. The SEQ is composed of three 8‐item subscales: academic self‐efficacy, social self‐efficacy, and emotional self‐efficacy. The SEQ measures responses using a 5‐point Likert scale ranging from 1 = strongly disagree to 5 = strongly agree, with higher scores indicating higher levels of self‐efficacy. The Cronbach's alpha in this sample was α = .82.

*Strengths and Difficulties Questionnaire (SDQ)* is a 25‐item questionnaire to assess mental health problems in children ages 11–17 (Goodman, 1997). The questionnaire assesses five sub‐dimensions including conduct problems, hyperactivity‐inattention, emotional symptoms, peer problems and prosocial behavior. Goodman et al. (2010) recommend use of broader internalizing and externalizing subscales of the SDQ in population screenings, with conduct problems and hyperactivity‐inattention subscales summed to create an “externalizing symptoms” subscale; and emotional symptoms and peer problems summed to create an “internalizing symptoms” subscale. Higher scores are indicative of greater mental health symptom levels. Response categories include three answer choices (0 = not true; 1 = somewhat true; 2 = certainly true). The Cronbach's alpha in this sample was α = .73.

*Additional Measures* included sex, age, class and school. Location of schools was used to determine urban, peri‐urban or rural communities from which the analytical sample was drawn.

## 3 RESULTS

Data analysis was performed using Stata version 17 (StataCorp, 2021). There was minimal missing data in the sample for measured variables (1.4%), and analyses proceeded with complete cases (*n* = 274). Demographic characteristics of the analytical sample are presented in Table 1. To evaluate the first study aim, descriptive statistics of key variables were assessed for significant differences by sex (Table 2). Compared to boys, girls reported higher scores on the AQ subscales for attention switching (*t* = 5.23; *p* = .005) and communication (*t* = 3.11; *p* = .002), and lower scores for imagination (*t* = −3.02, *p* = .003). On the SEQ, boys reported higher emotional self‐efficacy (*t* = −2.42; *p* = .016), social self‐efficacy (*t* = −3.15, *p* = .002), and total self‐efficacy (*t* = −2.24, *p* = .026). Girls reported higher total internalizing symptoms compared to boys (*t* = 5.10, *p* < .001). Associations between sociodemographic variables, autism trait subscales and total AQ scores, self‐efficacy subscales and total SEQ, and internalizing symptoms using Pearson's correlations are presented in Table 3. Age was positively associated with the AQ social skills subscale (ρ = 0.21, *p* < .010), with higher AQ social skills scores indicating greater difficulties. Age was also positively associated with total AQ score (ρ = 0.16, *p* < .010). Age was negatively correlated with academic self‐efficacy (ρ = −0.24, *p* < .001), social self‐efficacy (ρ = −0.15, *p* < .050), and total SEQ (ρ = −0.19, *p* < .010). Internalizing symptoms were negatively associated with male sex (ρ = −0.22, *p* < .001) and emotional self‐efficacy (ρ = −0.14, *p* < .050) and positively associated with AQ subscale scores for social skills (ρ = 0.16, *p* < .050), attention switching (ρ = 0.16, *p* < .01), and communication (ρ = 0.30, *p* < .001). Internalizing symptoms were also positively correlated with total AQ (ρ = 0.24, *p* < .010).

**TABLE 1:** Descriptive statistics of the analytical sample (*N* = 274).

Sex	*N* (%)
Female	135 (59.3)
Male	139 (50.7)
Age	
10	24 (8.8)
11	55 (20.1)
12	59 (21.5)
13	60 (21.9)
14	76 (27.7)
Mean (*SD*)	12.4 (1.3)
Class	
5	41 (15.0)
6	56 (20.5)
7	52 (19.1)
8	66 (24.2)
9	44 (16.1)
10	14 (5.3)
Location	
Urban	19 (6.9)
Peri‐urban	57 (20.8)
Rural	198 (72.3)

**TABLE 2:** Descriptive statistics of measured variables compared by gender.

	Female (*n* = 135)		Male (*n* = 139)				Total (*n* = 274)	
	Mean	*SD*	Mean	*SD*	*t*	*p* Value	Mean	*SD*
AQ								
Social Skills	3.46	2.07	3.07	1.79	1.67	.097	3.2	1.94
Attention Switching	5.23	1.52	4.70	1.56	2.86	.005**	5.0	1.56
Attention to Detail	5.90	1.85	5.96	1.87	–0.30	.763	5.9	1.85
Communication	4.14	1.72	3.50	1.71	3.11	.002**	3.8	1.74
Imagination	3.70	1.40	4.26	1.64	–3.02	.003**	4.0	1.55
Total AQ	22.21	4.23	21.26	4.16	1.85	.066	21.9	2.29
SEQ								
Emotional	29.1	0.49	30.7	0.42	–2.42	.016*	29.9	0.32
Social	30.3	0.40	32.1	0.41	–3.15	.002**	31.2	0.29
Academic	30.9	0.41	30.9	0.44	0.06	.956	30.9	0.30
Total SEQ	90.3	1.05	93.6	1.04	–2.24	.026*	92.0	0.75
SDQ								
Internalizing symptoms	8.0	0.31	5.9	0.28	5.10	.000***	6.9	0.22

AQ, Autism Spectrum Quotient; SEQ, Self‐Efficacy Questionnaire for Children; SDQ, Strengths and Difficulties Questionnaire.

*p < .05.

**p < .01.

***p < .001.

**TABLE 3:** Pearson's correlation coefficients for key analytic variables.

Key variables	1	2	3	4	5	6	7	8	9	10	11	12
1. Sex	–											
2. Age	–.05	–										
3. Social Skills	–.10	.21***	–									
4. Attention Switching	–.17**	.06	.23***	–								
5. Attention to Detail	.02	–.05	–.22***	.01	–							
6. Communication	–.19**	.07	.31***	.23***	–.12*	–						
7. Imagination	.18**	.08	.18**	.04	–.19**	.15*	–					
8. Total AQ	–.10	.16**	.63***	.58***	.22**	.63***	.44***	–				
9. Academic Self Efficacy	–.00	–.24***	–.17**	–.07	.21***	–.10	–.06	–.09	–			
10. Social Self Efficacy	.19**	–.15*	–.41***	–.15*	.11	–.23**	–.13*	–.32***	.44***	–		
11. Emotional Self Efficacy	.15*	–.08	–.33***	–.13*	.17**	–.18**	–.07	–.21***	.47***	.60***	–	
12. Total SEQ	.14*	–.19**	–.37***	–.14*	.20***	–.21***	–.10	–.25***	.77***	.82***	.85***	–
13. Internalizing Symptoms	–.22***	–.087	.16*	.16**	–.023	.30***	.01	.24***	.07	–.08	–.14*	–.06

*p < .05.

**p < .01.

***p < .001.

To evaluate the second study aim, three mixed‐effect regression models were executed to assess the relationship between independent variables on the dependent variable, internalizing symptoms. Each model was adjusted for sex (Male = 2; Female = 1), age, and for clustering within schools, with robust standard errors reported (Table 4). *Model 1* included the academic, social and emotional subscales of the SEQ. *Model 2* included the social skills, attention switching, attention to detail, communication, and imagination subscales of the AQ. *Model 3* included the total SEQ and total AQ score, and the interaction term between total SEQ and total AQ. *Model 1* accounted for 8% of the variation in internalizing symptoms. Males reported significantly lower internalizing symptoms (β = −1.30, *p* < .001) compared to females. Youth with higher emotional SEQ scores reported significantly lower internalizing symptoms (β = −0.10, *p* = .005), whereas youth with higher academic SEQ scores (β = 0.09, *p* = .023) had significantly higher internalizing symptoms. *Model 2* accounted for 13% of the variation in internalizing symptoms. Consistent with *Model 1*, male sex (β = −0.97, *p* < .001) was associated with lower internalizing symptoms. Among the AQ subscales, only the communication subscale (β = 0.45, *p* = .008) was associated with higher internalizing symptoms. *Model 3* accounted for 12% of the variation in internalizing symptoms. Again, male sex (β = −0.121, *p* < .001) was associated with lower internalizing symptoms. The total SEQ score was associated with higher internalizing symptoms (β = 0.14, *p* < .001), as was the total AQ score (β = 0.74, *p* < .001). The interaction term between total SEQ and total AQ was significant, though with a small effect size (β = −0.01, *p* < .001).

**TABLE 4:** Multivariate regression models predicting internalizing symptoms.

	Internalizing symptoms (SDQ)				
	β	*SE*	*t*	*p*	*R* ^2^
*Model 1: SEQ*					.08
Age	–0.20	0.29	–0.68	.495	
Sex	–1.30	0.23	–5.68	<.001	
Academic SEQ	0.09	0.04	2.28	.023	
Social SEQ	–0.13	0.07	–0.21	.837	
Emotional SEQ	–0.10	0.04	–2.78	.005	
*Model 2: AQ*					.13
Age	–0.32	0.29	–1.12	.263	
Sex	–0.97	0.28	–3.50	<.001	
Social Skills	0.10	0.06	1.61	.108	
Attention Switching	0.18	0.11	1.60	.110	
Attention Detail	0.05	0.13	0.38	.701	
Communication	0.43	0.16	2.66	.008	
Imagination	0.04	0.12	0.35	.728	
*Model 3: SEQ × AQ*					.12
Age	–0.35	0.28	–1.26	.208	
Sex	–1.21	0.29	–4.25	<.001	
Total SEQ	0.14	0.04	3.82	<.001	
Total AQ	0.74	0.16	4.51	<.001	
SEQ × AQ	–0.01	0.01	–3.85	< .001	

SEQ, Self‐Efficacy Questionnaire for Children; AQ, Autism Quotient; SDQ, Strengths and Difficulties Questionnaire.

*p ≤ .05.

**p < .01.

***p ≤ .001.

**FIGURE 1:**
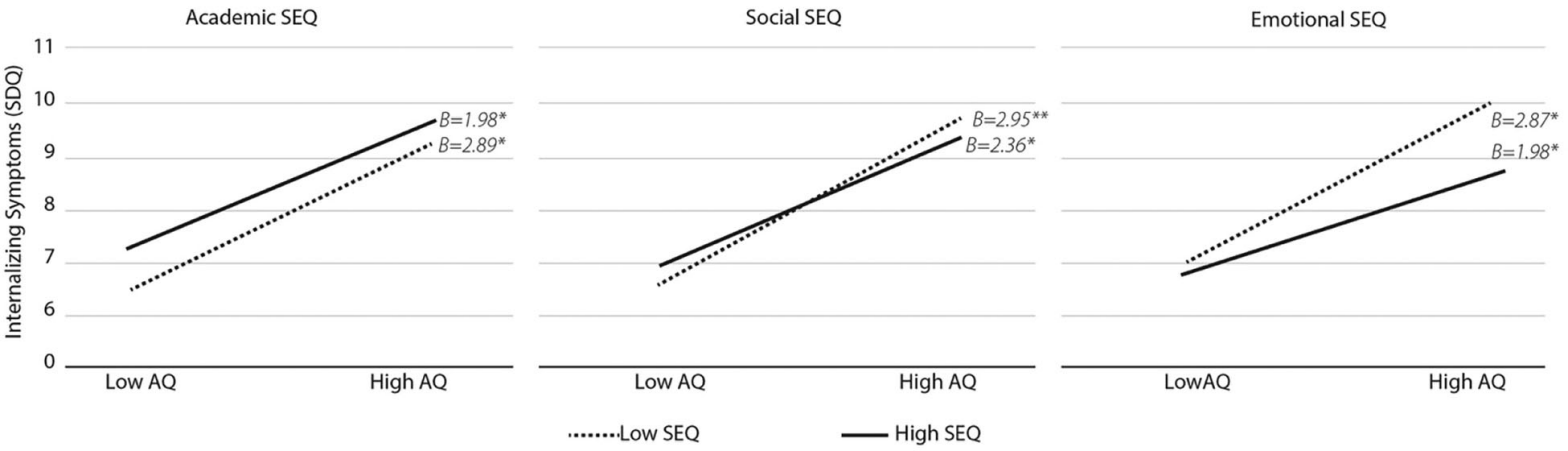
Marginal interaction plot of dimensions of self‐efficacy and autism traits to predict internalizing symptoms. Interaction plotted using autism traits as measured by the total score on the Autism Spectrum Questionnaire (low and high AQ reflect 1 standard deviation below and above the mean, respectively); the total scores on dimensions of the Self‐Efficacy Questionnaire for Children (low and high SEQ represent 1 standard deviation below and above the mean for each dimension, respectively); internalizing symptoms are measured by the Strengths and Difficulties Questionnaire.

Lastly, a marginal interaction model was fitted between academic, social and emotional subscales of the SEQ and autism traits (low: 1 standard deviation below the mean, high: 1 standard deviation above the mean) for the dependent variable of internalizing symptoms Figure 1. It was found that individuals with higher academic self‐efficacy showed elevated internalizing symptoms regardless of their AQ scores. Conversely, those with high social self‐efficacy and high AQ scores exhibited lower internalizing symptoms compared to individuals with low social self‐efficacy and high AQ scores. Similarly, individuals with both low and high AQ scores showed reduced internalizing symptoms when they had higher emotional self‐efficacy scores, compared to those with lower emotional self‐efficacy scores.

## 4 DISCUSSION

The current study extends research on the associations between autistic traits and internalizing symptoms, which may be moderated by academic, social, and emotional subdomains of self‐efficacy, in a nonclinical sample of early adolescents from a LMIC. Consistent with well‐established global findings, boys reported significantly lower internalizing symptoms compared to girls in all models. In contrast to other studies, there were no differences in total AQ scores by sex in this sample; however, sex differences were observed in AQ subscales. Specifically, girls reported higher scores on the attention switching and communication subscales, and lower scores on the imagination subscale.

In the multivariate regression models, higher scores on the communication subscale were associated with greater internalizing symptoms. Difficulties with communication among autistic and neurodiverse individuals have been associated with social rejection and experiences of stigma, contributing to internalizing symptoms (Botha et al., 2022). This finding suggests that interventions designed to support neurodivergent early adolescents should consider enhancing communication skills, particularly for girls. Strategies could include encouraging the acquisition of verbal communication skills, as well as using alternative communication strategies such as written communication, recognizing gestures and signing, or supporting use of augmentative and alternative communication (AAC) (Cariveau et al., 2022; Holyfield et al., 2017). Furthermore, while individualized approaches to improving communication are useful for tailoring to the unique strengths and challenges of autistic youth, there is a need to better understand how school climate and contextual factors could be targeted through strengths‐based programing to support neurodivergent youth with communication differences. For example, incorporating visual schedules in classrooms, simplified directions in assignments, dividing assignments in to smaller sections, or flexibility in demonstrating knowledge acquisition (written, verbal, or nonverbal) could enhance individuals’ self‐efficacy while providing opportunities to showcase their competencies.

In this sample, girls reported lower social and emotional self‐efficacy, as well as lower total self‐efficacy compared to boys. This finding aligns with previous research in nonclinical American and Tanzanian adolescent samples (Cherewick et al., 2023; Muris, 2001). In the present study, academic self‐efficacy was significantly associated with higher internalizing symptoms. Academic self‐efficacy refers to the individual's perceived ability to control their learning behaviors, master subjects, and meet scholastic expectancies (Muris, 2001). In the Indian context, extensive research has indicated that youth report enormous stress related to academic pressure (Khan et al., 2016; Kumar & Talwar, 2014; Mathew et al., 2015). The positive association between academic self‐efficacy and internalizing symptoms found in this study may be influenced by adolescent stress levels. It is plausible that adolescents with greater academic self‐efficacy also experience higher levels of stress. Specifically, for youth at both the high and low end of the AQ scales, higher academic self‐efficacy was associated with increased internalizing symptoms.

In contrast to academic self‐efficacy, both higher social and emotional self‐efficacy reduced internalizing symptoms among youth scoring higher on the AQ scale, compared to those scoring lower. Consistent with previous studies linking emotional self‐efficacy to lower psychological symptoms, this study identified emotional self‐efficacy as having the strongest protective association against internalizing symptoms among youth with higher levels of autistic traits (Andretta & McKay, 2020). A noteworthy finding in this sample was the moderating effect of social self‐efficacy on internalizing symptoms between high and low autism trait scores. This finding aligns with research on social connectedness and loneliness, which partially explains the association between autistic traits and internalizing symptoms (Schiltz et al., 2021; Stice & Lavner, 2019). The strong correlation observed in this sample between social and emotional self‐efficacy, as well as between AQ subscales and social and emotional self‐efficacy, suggests that *social‐emotional learning* interventions for neurodivergent youth could be a promising approach to reducing internalizing symptoms during early adolescence, before they potentially reach clinically significant levels in mid to later adolescence. Previous studies on social skills interventions have demonstrated reductions in internalizing symptoms (McVey et al., 2016; Schiltz et al., 2018). Integrating content that enhances emotional self‐efficacy through effective emotional regulation and expression, in conjunction with social self‐efficacy content, can leverage the protective effects of both domains on internalizing symptoms. These interventions should consider utilizing nonspecialists outside of the school context (e.g. near‐peers, community health workers) to differentiate the social‐emotional learning program from academic learning and to mitigate existing stress associated with the academic environment.

Limitations of the study include the potential risk of bias resulting from self‐report by adolescents on the survey measures. Future research should consider triangulating findings by having multiple participants such as teachers and caregivers to assess early adolescents. Moreover, research suggests that there may be differences in perceived self‐efficacy between more collectivistic versus individualistic cultures (Bond, 1991), which may limit the generalizability of study findings to other contexts. Additional measurement considerations should include scales that capture broader dimensions of neurodiversity and learning to assess associations with internalizing symptoms more comprehensively. Furthermore, measures of perceived stress and social connectedness could help reduce potential confounding in the relationship between autistic traits, self‐efficacy, and internalizing symptoms. Lastly, this study utilized a cross‐sectional sample of early adolescents. Future studies should consider longitudinal data collection to test developmental trajectories in reported autistic traits, self‐efficacy, and psychological symptoms, which could enhance the timing and precision of intervention delivery and design.

In conclusion, this study significantly contributes to an expanding literature on neurodiversity and autism by uncovering associations between autistic traits, dimensions of self‐efficacy, and internalizing symptoms in a nonclinical sample of early adolescents from a LMIC. Our findings suggest the need to develop social‐emotional learning interventions for neurodivergent youth to effectively mitigate internalizing symptoms and improve mental health trajectories.

## AUTHOR CONTRIBUTIONS

**Megan Cherewick**: Conceptualization; methodology; formal analysis; supervision; project administration; funding acquisition; writing—original draft; writing—review and editing. **Christina M. Cruz**: Conceptualization; writing—review and editing. **Priscilla Giri**: Writing—review and editing; conceptualization; methodology; data curation; investigation; supervision. **Michael Matergia**: Writing—review and editing; conceptualization; supervision; project administration; investigation. **Roshan P. Rai**: Conceptualization; writing—review and editing; supervision; project administration.

## FUNDING

The authors received no financial support for the research, authorship, or publication of this article.

## CONFLICT OF INTEREST STATEMENT

The authors have no relevant financial or nonfinancial interests to disclose.

### PEER REVIEW

The peer review history for this article is available at https://publons.com/publon/10.1002/brb3.3646.

## Data Availability

The data that support the findings of this study are available on request from the corresponding author. The data are not publicly available due to privacy or ethical restrictions.

## References

[brb33646-bib-0001] Andretta, J. R. , & McKay, M. T. (2020). Self‐efficacy and well‐being in adolescents: A comparative study using variable and person‐centered analyses. Children and Youth Services Review, 118, 105374. 10.1016/j.childyouth.2020.105374

[brb33646-bib-0002] Baird, G. , Simonoff, E. , Pickles, A. , Chandler, S. , Loucas, T. , Meldrum, D. , & Charman, T. (2006). Prevalence of disorders of the autism spectrum in a population cohort of children in South Thames: The Special Needs and Autism Project (SNAP). The Lancet, 368(9531), 210–215. 10.1016/S0140-6736(06)69041-7 16844490

[brb33646-bib-0003] Bandura, A. , Freeman, W. H. , & Lightsey, R. (1999). Self‐efficacy: The exercise of control. Springer.

[brb33646-bib-0004] Baron‐Cohen, S. , Wheelwright, S. , Skinner, R. , Martin, J. , & Clubley, E. (2001). The autism‐spectrum quotient (AQ): Evidence from Asperger syndrome/high‐functioning autism, males and females, scientists and mathematicians. Journal of Autism and Developmental Disorders, 31(1), 5–17. 10.1023/a:1005653411471 11439754

[brb33646-bib-0005] Bond, M. H. (1991). Beyond the Chinese face: Insights from psychology. Oxford University Press.

[brb33646-bib-0006] Botha, M. , Dibb, B. , & Frost, D. M. (2022). ‘It's being a part of a grand tradition, a grand counter‐culture which involves communities’: A qualitative investigation of autistic community connectedness. Autism, 26(8), 2151–2164. 10.1177/13623613221080248 35318862 PMC9597163

[brb33646-bib-0007] Broadbent, J. , Galic, I. , & Stokes, M. A. (2013). Validation of autism spectrum quotient adult version in an Australian sample. Autism Research and Treatment, 2013, 984205. 10.1155/2013/984205 23762552 PMC3665170

[brb33646-bib-0008] Cariveau, T. , Hunt, K. , Robbins, H. , & Brown, A. R. (2022). Preference for alternative communication modality based on reinforcer quality and availability. Behavior Modification, 46(4), 799–818. 10.1177/0145445521992308 33538179

[brb33646-bib-0009] Cherewick, M. (2023). Future directions for neurodiversity affirming autism interventions: Adolescence is a second sensitive period to support positive developmental trajectories. Current Psychology, 43, 9191–9198. 10.1007/s12144-023-05053-7

[brb33646-bib-0010] Cherewick, M. , Hipp, E. , Njau, P. , & Dahl, R. E. (2023). Growth mindset, persistence, and self‐efficacy in early adolescents: Associations with depression, anxiety, and externalising behaviours. Global Public Health, 18(1), 2213300. 10.1080/17441692.2023.2213300 37196667

[brb33646-bib-0011] Comunian, A. L. (1989). Some characteristics of relations among depression, anxiety, and self‐efficacy. Perceptual and Motor Skills, 69(3‐1), 755–764. 10.2466/pms.1989.69.3.755 2608390

[brb33646-bib-0012] Deshmukh, V. , Mohapatra, A. , Gulati, S. , Nair, M. , Bhutani, V. , & Silberg, D. (2013). Prevalence of neuro‐developmental disorders in India: Poster presentation. *Program booklet*. West Hartford: IMFAR, 76.

[brb33646-bib-0013] Ehrenberg, M. F. , Cox, D. N. , & Koopman, R. F. (1991). The relationship between self‐efficacy and depression in adolescents. Adolescence, 26(102), 361. 1927668

[brb33646-bib-0014] Fombonne, E. (2009). Epidemiology of pervasive developmental disorders. Pediatric Research, 65(6), 591–598. 10.1203/PDR.0b013e31819e7203 19218885

[brb33646-bib-0015] Goodman, A. , Lamping, D. L. , & Ploubidis, G. B. (2010). When to use broader internalising and externalising subscales instead of the hypothesised five subscales on the Strengths and Difficulties Questionnaire (SDQ): Data from British parents, teachers and children. Journal of Abnormal Child Psychology, 38, 1179–1191. 20623175 10.1007/s10802-010-9434-x

[brb33646-bib-0016] Goodman, R. (1997). The strengths and difficulties questionnaire: A research note. Journal of Child Psychology and Psychiatry, 38(5), 581–586. 9255702 10.1111/j.1469-7610.1997.tb01545.x

[brb33646-bib-0017] Hodgetts, S. , Zwaigenbaum, L. , & Nicholas, D. (2015). Profile and predictors of service needs for families of children with autism spectrum disorders. Autism, 19(6), 673–683. 10.1177/1362361314543531 25073749 PMC4509871

[brb33646-bib-0018] Hoekstra, R. A. , Bartels, M. , Cath, D. C. , & Boomsma, D. I. (2008). Factor structure, reliability and criterion validity of the Autism‐Spectrum Quotient (AQ): A study in Dutch population and patient groups. Journal of Autism and Developmental Disorders, 38(8), 1555–1566. 10.1007/s10803-008-0538-x 18302013 PMC2516538

[brb33646-bib-0019] Holyfield, C. , Drager, K. D. R. , Kremkow, J. M. D. , & Light, J. (2017). Systematic review of AAC intervention research for adolescents and adults with autism spectrum disorder. Augmentative and Alternative Communication (Baltimore, Md.: 1985), 33(4), 201–212. 10.1080/07434618.2017.1370495 28884601

[brb33646-bib-0020] Johnson, M. H. (2017). Autism as an adaptive common variant pathway for human brain development. Developmental Cognitive Neuroscience, 25, 5–11. 10.1016/j.dcn.2017.02.004 28233663 PMC6987822

[brb33646-bib-0021] Kashdan, T. B. , & Roberts, J. E. (2004). Social anxiety's impact on affect, curiosity, and social self‐efficacy during a high self‐focus social threat situation. Cognitive Therapy and Research, 28(1), 119–141. 10.1023/B:COTR.0000016934.20981.68

[brb33646-bib-0022] Khan, A. , Hamdan, A. R. , Ahmad, R. , Mustaffa, M. S. , & Mahalle, S. (2016). Problem‐solving coping and social support as mediators of academic stress and suicidal ideation among Malaysian and Indian adolescents. Community Mental Health Journal, 52(2), 245–250. 26499062 10.1007/s10597-015-9937-6

[brb33646-bib-0023] Kogan, M. D. , Strickland, B. B. , Blumberg, S. J. , Singh, G. K. , Perrin, J. M. , & van Dyck, P. C. (2008). A national profile of the health care experiences and family impact of autism spectrum disorder among children in the United States, 2005–2006. Pediatrics, 122(6), e1149–e1158. 10.1542/peds.2008-1057 19047216

[brb33646-bib-0024] Kumar, V. , & Talwar, R. (2014). Determinants of psychological stress and suicidal behavior in Indian adolescents: A literature review. Journal of Indian Association for Child and Adolescent Mental Health, 10(1), 47–68.

[brb33646-bib-0025] Kwasky, A. N. , & Groh, C. J. (2014). Vitamin D, depression and coping self‐efficacy in young women: Longitudinal study. Archives of Psychiatric Nursing, 28(6), 362–367. 10.1016/j.apnu.2014.08.010 25457684

[brb33646-bib-0026] Lai, M.‐C. , Lombardo, M. V. , Auyeung, B. , Chakrabarti, B. , & Baron‐Cohen, S. (2015). Sex/gender differences and autism: Setting the scene for future research. Journal of the American Academy of Child & Adolescent Psychiatry, 54(1), 11–24. 25524786 10.1016/j.jaac.2014.10.003 PMC4284309

[brb33646-bib-0027] Lau, W. Y. , Kelly, A. B. , & Peterson, C. C. (2013). Further evidence on the factorial structure of the Autism Spectrum Quotient (AQ) for adults with and without a clinical diagnosis of autism. Journal of Autism and Developmental Disorders, 43(12), 2807–2815. 10.1007/s10803-013-1827-6 23619950

[brb33646-bib-0028] Lord, C. , & Spence, S. J. (2006). Autism spectrum disorders: Phenotype and diagnosis. In S. O. Moldin & J. L. R. Rubenstein (Eds.), Understanding autism: From basic neuroscience to treatment (pp. 1–23). CRC Press/Routledge/Taylor & Francis Group.

[brb33646-bib-0029] Luszczynska, A. , Gutiérrez‐Doña, B. , & Schwarzer, R. (2005). General self‐efficacy in various domains of human functioning: Evidence from five countries. International Journal of Psychology, 40(2), 80–89. 10.1080/00207590444000041

[brb33646-bib-0030] Lyall, K. , Croen, L. , Daniels, J. , Fallin, M. D. , Ladd‐Acosta, C. , Lee, B. K. , Park, B. Y. , Snyder, N. W. , Schendel, D. , & Volk, H. (2017). The changing epidemiology of autism spectrum disorders. Annual Review of Public Health, 38, 81–102. 10.1146/annurev-publhealth-031816-044318 PMC6566093 28068486

[brb33646-bib-0031] Mathew, N. , Khakha, D. C. , Qureshi, A. , Sagar, R. , & Khakha, C. C. (2015). Stress and coping among adolescents in selected schools in the capital city of India. The Indian Journal of Pediatrics, 82(9), 809–816. 10.1007/s12098-015-1710-x 25689960

[brb33646-bib-0032] McVey, A. J. , Dolan, B. K. , Willar, K. S. , Pleiss, S. , Karst, J. S. , Casnar, C. L. , Caiozzo, C. , Vogt, E. M. , Gordon, N. S. , & Van Hecke, A. V. (2016). A replication and extension of the PEERS® for young adults social skills intervention: Examining effects on social skills and social anxiety in young adults with autism spectrum disorder. Journal of Autism and Developmental Disorders, 46(12), 3739–3754. 10.1007/s10803-016-2911-5 27628940 PMC5310211

[brb33646-bib-0033] Milton, D. , & Sims, T. (2016). How is a sense of well‐being and belonging constructed in the accounts of autistic adults? Disability & Society, 31(4), 520–534. 10.1080/09687599.2016.1186529

[brb33646-bib-0034] Mottron, L. , Belleville, S. , Rouleau, G. A. , & Collignon, O. (2014). Linking neocortical, cognitive, and genetic variability in autism with alterations of brain plasticity: The Trigger‐Threshold‐Target model. Neuroscience & Biobehavioral Reviews, 47, 735–752. 10.1016/j.neubiorev.2014.07.012 25155242

[brb33646-bib-0035] Muris, P. (2001). A brief questionnaire for measuring self‐efficacy in youths. Journal of Psychopathology and Behavioral Assessment, 23(3), 145–149. 10.1023/A:1010961119608

[brb33646-bib-0036] Patridge, E. F. , & Bardyn, T. P. (2018). Research electronic data capture (REDCap). Journal of the Medical Library Association: JMLA, 106(1), 142.

[brb33646-bib-0037] Rahman, A. , Divan, G. , Hamdani, S. , Vajaratkar, V. , Taylor, C. , Leadbitter, K. , & Green, J. (2016). The effectiveness of the parent‐mediated intervention for autism spectrum disorders in South Asia (PASS): A randomised controlled trial in India and Pakistan. Lancet Psychiatry, 3(2), 128–136. 26704571 10.1016/S2215-0366(15)00388-0

[brb33646-bib-0038] Sandin, B. , Sánchez‐Arribas, C. , Chorot, P. , & Valiente, R. M. (2015). Anxiety sensitivity, catastrophic misinterpretations and panic self‐efficacy in the prediction of panic disorder severity: Towards a tripartite cognitive model of panic disorder. Behaviour Research and Therapy, 67, 30–40. 10.1016/j.brat.2015.01.005 25727680

[brb33646-bib-0039] Schiltz, H. K. , McVey, A. J. , Dolan, B. K. , Willar, K. S. , Pleiss, S. , Karst, J. S. , Carson, A. M. , Caiozzo, C. , Vogt, E. M. , Yund, B. D. , & Van Hecke, A. V. (2018). Changes in depressive symptoms among adolescents with ASD completing the PEERS(®) social skills intervention. Journal of Autism and Developmental Disorders, 48(3), 834–843. 10.1007/s10803-017-3396-6 29164445 PMC10321229

[brb33646-bib-0040] Schiltz, H. K. , McVey, A. J. , Dolan Wozniak, B. , Haendel, A. D. , Stanley, R. , Arias, A. , Gordon, N. , & Van Hecke, A. V. (2021). The role of loneliness as a mediator between autism features and mental health among autistic young adults. Autism, 25(2), 545–555. 10.1177/1362361320967789 33126822

[brb33646-bib-0041] Schönfeld, P. , Brailovskaia, J. , Bieda, A. , Zhang, X. C. , & Margraf, J. (2016). The effects of daily stress on positive and negative mental health: Mediation through self‐efficacy. International Journal of Clinical and Health Psychology, 16(1), 1–10. 10.1016/j.ijchp.2015.08.005 30487845 PMC6225043

[brb33646-bib-0042] StataCorp . (2021). Stata Statistical Software: Release 17. StataCorp, LLC.

[brb33646-bib-0043] Stice, L. V. , & Lavner, J. A. (2019). Social connectedness and loneliness mediate the association between autistic traits and internalizing symptoms among young adults. Journal of Autism and Developmental Disorders, 49(3), 1096–1110. 10.1007/s10803-018-3812-6 30406911

[brb33646-bib-0044] Vohra, R. , Madhavan, S. , & Sambamoorthi, U. (2017). Comorbidity prevalence, healthcare utilization, and expenditures of Medicaid enrolled adults with autism spectrum disorders. Autism, 21(8), 995–1009. 27875247 10.1177/1362361316665222 PMC5517354

